# Implications of Aperiodic and Periodic EEG Components in Classification of Major Depressive Disorder from Source and Electrode Perspectives

**DOI:** 10.3390/s24186103

**Published:** 2024-09-21

**Authors:** Ahmad Zandbagleh, Saeid Sanei, Hamed Azami

**Affiliations:** 1 School of Electrical Engineering, Iran University of Science and Technology, Tehran 16846-13114, Iran; a_zandbagleh@alumni.iust.ac.ir; 2 Electrical and Electronic Engineering Department, Imperial College London, London SW7 2AZ, UK; s.sanei@imperial.ac.uk; 3 Centre for Addiction and Mental Health, University of Toronto, Toronto, ON M6J 1H1, Canada

**Keywords:** EEG, machine learning, major depressive disorder, power spectral density, periodic and aperiodic components

## Abstract

Electroencephalography (EEG) is useful for studying brain activity in major depressive disorder (MDD), particularly focusing on theta and alpha frequency bands via power spectral density (PSD). However, PSD-based analysis has often produced inconsistent results due to difficulties in distinguishing between periodic and aperiodic components of EEG signals. We analyzed EEG data from 114 young adults, including 74 healthy controls (HCs) and 40 MDD patients, assessing periodic and aperiodic components alongside conventional PSD at both source and electrode levels. Machine learning algorithms classified MDD versus HC based on these features. Sensor-level analysis showed stronger Hedge’s g effect sizes for parietal theta and frontal alpha activity than source-level analysis. MDD individuals exhibited reduced theta and alpha activity relative to HC. Logistic regression-based classifications showed that periodic components slightly outperformed PSD, with the best results achieved by combining periodic and aperiodic features (AUC = 0.82). Strong negative correlations were found between reduced periodic parietal theta and frontal alpha activities and higher scores on the Beck Depression Inventory, particularly for the anhedonia subscale. This study emphasizes the superiority of sensor-level over source-level analysis for detecting MDD-related changes and highlights the value of incorporating both periodic and aperiodic components for a more refined understanding of depressive disorders.

## 1. Introduction

Major depressive disorder (MDD) is one of the most prevalent mental health disorders, characterized by persistent depressed mood, diminished interest, recurrent thoughts of death or suicide, and impaired physical and cognitive function [1,2]. MDD is a leading cause of functional disability worldwide, with approximately 300 million individuals reported to be affected by this disorder in 2015 [3]. The diagnosis of MDD is difficult due to the lack of established biomarkers and the subjective nature of patient responses to psychological evaluations [4].

Individuals with MDD often exhibit abnormal patterns of brain activity when compared to healthy individuals [1,2,5,6,7]. The advancement of non-invasive electroencephalography (EEG) technology has paved the way and become a valuable tool for studying brain activity due to its low cost, portability, and high temporal resolution [8]. A number of studies using resting-state EEGs have shown altered brain activity in MDD using power spectral density (PSD) [4,5]. According to the literature, while alterations in some frequency bands have been observed, changes in the power of theta and alpha frequency bands are more commonly documented [4,5,9,10].

Research findings support that when individuals experience emotional arousal, neurons in the amygdala generate theta waves [11,12]. This suggests a link between theta brain activity and emotions [13]. Additionally, findings imply that hippocampal theta activity plays a role in distinguishing emotional stimuli [13]. A disruption in emotional processing is a characteristic feature of depression [14,15,16], and alterations in theta oscillations might indicate irregularities in how individuals with MDD process and regulate emotions. Alpha oscillations indicate relaxation and lack of brain activity, playing a role in inhibitory functions that are linked to various cognitive processes, such as working memory, attention, and emotional regulation [13,17]. On the other hand, glutamate is the main excitatory neurotransmitter in the brain, and disruptions in its transmission have consistently been linked to the development of MDD [18]. Furthermore, decreased cortical inhibition is a key factor in the process of depression [19]. The primary inhibitory neurotransmitter, known as gamma-aminobutyric acid (GABA), plays a crucial role in regulating emotions within the limbic regions [19,20,21]. Converging evidence indicates that in individuals with MDD, GABA levels are notably lower compared to healthy individuals [18]. The interactions among glutamatergic and GABAergic (as well as cholinergic and serotonergic) receptors in the thalamus and cortex play a critical role in regulating alpha power and frequency, which are essential aspects of neural activity modulation [22]. In sum, these findings highlight the importance of further research into the theta and alpha frequency bands for this disorder.

Numerous studies have examined the significance of theta and alpha frequency bands in individuals with MDD [4,5,23,24,25,26,27,28,29,30,31]. One study [23] suggested that theta and alpha frequency bands effectively distinguish between normal and MDD individuals, aligning with prior research that associates these bands with emotional processing [15,16]. Some other studies [24,25] demonstrated that alpha power serves as a valuable predictor of depression. Delson et al. [26] showed that increased alpha activity during the entire night’s sleep is associated with higher levels of suicidal thoughts in people with depression. Some studies have reported an increase [27,28] in relative alpha power, particularly in the frontal [27,28], parietal [27] and parietotemporal [28] brain regions. Furthermore, two studies [29,30] demonstrated an increase in power of theta and alpha frequency bands in parietal and occipital brain areas, suggesting reduced cortical activation within these regions [30]. In contrast, one study [31] used 32-channel EEG and showed that patients with depression exhibited lower alpha waves compared to healthy control (HC) subjects in the frontal, parietal, temporal, and occipital lobes. Similarly, Lee et al. demonstrated a decrease in the relative alpha power among individuals with depression at different EEG electrodes in frontal, temporal, central, and occipital regions [25]. Although some studies have demonstrated the association between the treatment response and the increased theta activations [5], there are conflicting results in pre-treatment and early changes in the theta band. Some studies suggest a decrease in theta activity after treatment [32,33], while others propose an association between increased theta activation and the treatment response [34].

However, findings related to the power of theta and alpha frequency bands in different brain regions for individuals with MDD compared to healthy individuals have not been consistently replicated [4,5,9]. One possible explanation for the inconsistencies in these studies could be the failure in removing the aperiodic component of EEG signals. Notably, EEG signals comprise both the arrhythmic background part of the spectrum (referred to as the 1/*f* noise-like component) and rhythmic neural oscillations (the periodic component) [35]. The aperiodic component of EEG, often referred to as fractal or “scale-free” activity, exhibits self-similar patterns across various temporal scales [36]. Previous research has emphasized the importance of concentrating on the periodic components of EEG signals, which have been linked to the processing speed and the working memory, rather than the aperiodic components [35,37,38,39]. Removing aperiodic components from EEG signals might be beneficial because they can mask subtle changes in true the oscillatory power in which may be present in MDD [35].

To the best of our knowledge, the impact of excluding the aperiodic component of resting-state EEG in both electrode and source levels has not been investigated in individuals with MDD. The electrode level refers to signals recorded directly from the scalp, whereas the source level involves identifying the specific brain regions responsible for the activity detected on the scalp [40]. In this study, our aim is to determine whether distinguishing individuals with MDD from HC could be improved by utilizing periodic and aperiodic components, as opposed to considering the full spectrum across both electrode and source levels. Furthermore, we used the most significant regions and frequency bands of periodic and aperiodic components, as well as PSD, to classify MDD and HC groups. Finally, for exploratory purposes, we calculated the correlation between these features and the Beck Depression Inventory (BDI) and its subscales, due to their importance in diagnosing depression. The main contributions of this study are as follows:

- Novel Investigation: This study is the first to investigate the impact of excluding the aperiodic component of resting-state EEG in different brain regions and in both electrode- and source-level EEGs for individuals with MDD vs. HC.
- Improved Classification: By utilizing periodic and aperiodic components separately, we aim to enhance the accuracy of distinguishing between MDD and HC individuals.
- Exploratory Analysis: We calculate the correlation between EEG features and the BDI and its subscales, providing additional insights into the neural correlates of depression.

## 2. Materials and Methods

### 2.1. Participants

In the current study, the publicly available dataset provided by Cavanagh et al. [41] originally contained 121 individuals (49 males, age range 18–25, mean = 18.86 ± 1.19). However, the practical information of two subjects was not available; therefore, they were removed from the dataset [42]. All participants were chosen from introductory psychology classes based on their scores on the BDI mass survey and provided informed consent, which was approved by the University of Arizona. For more detailed information about the participants, please refer to [42]. To ensure that only high-quality data were included in the final analysis, we excluded 5 MDD participants (3 males and 2 females) due to poor recording channel quality or insufficient data remaining after pre-processing. As a result, this study included 74 control participants with low BDI scores (<7) and 40 individuals diagnosed with depression who exhibited high BDI scores (≥13). Table 1 displays the demographic characteristics, as well as the scores derived from the BDI subscales and the Trait Anxiety Inventory (TAI).

### 2.2. EEG Data Recording and Processing

The resting-state EEG signal was obtained using the Synamps2 EEG system (Neuroscan, Inc., Charlotte, NC, USA), bandpass filtered using 0.5–100 Hz, with 64 Ag/AgCl EEG channels according to the international 10–20 system, and a sampling rate of 500 Hz [42]. Electrode impedances were maintained below 10 kΩ. All EEG pre-processing steps were carried out using the Harvard Automated Preprocessing Pipeline (HAPPE) [43] in EEGLAB toolbox [44] running on MATLAB 2019a (MathWorks, Inc., Natick, MA, USA). The HAPPE pipeline includes 1 Hz high-pass filtering, channel selection (removal of non-relevant electrode channels), 50 Hz grid noise removal, bad channel rejection, the use of a wavelet-enhanced independent component analysis (W-ICA) approach to correct EEG artifacts while maintaining the full length of the data, running the ICA extended infomax algorithm, and subsequent automatic component rejection using Multiple Artifact Rejection Algorithm (MARA) [45]. Bad channels are then interpolated, and the data are re-referenced to a common average reference. For further validation, after data segmentation (2 s), all segments are visually inspected.

**Figure 1:**
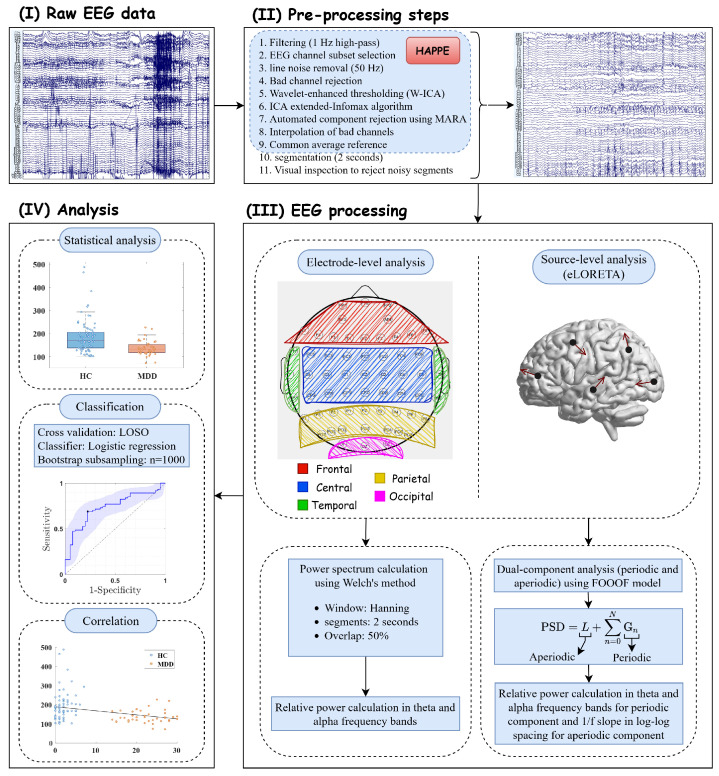
The flowchart of the overall proposed method: (**I**) resting-state EEG acquisition, (**II**) pre-processing steps, (**III**) EEG analysis using periodic and aperiodic components along with conventional PSD at both source and electrode levels, and (**IV**) statistical analysis, classification, and correlation analysis.

Welch’s method [46] (Hanning window function, 2-s segment size with a 50% overlap) and the median, without employing a scale factor, were used to carry out the robust spectral estimation [35]. The fooof (fitting of one over f) toolbox [35], which decomposes the power spectrum into periodic and aperiodic components, was used to parameterize the power spectrum. This toolbox uses a least-squared-error approach to optimize the modeled spectrum [35]. In our EEG data analysis, we utilized the frequency bands delta (1–4 Hz), theta (4–8 Hz), alpha (8–13 Hz), beta (13–30 Hz), and gamma (30–45 Hz). The algorithm employed in this study considers the PSD by separating it into an aperiodic background component and periodic oscillations, which are identified as frequency regions exhibiting power levels higher than those of the background component. In the PSD, the frequencies were linearly spaced, while the power values were log-spaced, and the algorithm operated in semilog-power space. The aperiodic component was modeled as a function across the spectrum, and a Gaussian was used to model each periodic component. Hence, the PSD was modeled as the sum of aperiodic component *L* and all the oscillatory Gaussian peaks *G*. This formulation represents the PSD as [35]

(1) $$\mathrm{PSD}=L+\sum_{n=0}^{N}G_{n}$$

where *L* represents the aperiodic component, and *N* denotes the total number of peaks identified from the power spectrum. Each $G_{n}$, which is defined as a Gaussian fitted to a peak, is represented as follows [35]:

(2) $$G_{n}=a\times exp\left(\frac{-{\left(F-C\right)}^{2}}{2w^{2}}\right)$$

where *a* represents the power of the peak value in logarithmic (base 10) scale, *F* denotes the vector of input frequencies, *C* indicates the center frequency in hertz (Hz), and *w* shows the standard deviation of the Gaussian in Hz. The aperiodic component, denoted as *L*, is represented by a Lorentzian function, formulated as [35]

(3) $$L=b-log(k+F^{\chi })$$

where *b* and χ represent the broadband offset and the exponent, respectively. The bend in the aperiodic component is controlled by the knee parameter, represented by *k*. When *k* is set to 0, this model corresponds to fitting a straight line in log-log space, also referred to as the fixed mode. It is important to note that there is a direct relationship between the slope, *a* in Equation (2), of the line in the log-log space and the exponent, χ. Therefore, when there is no knee, χ equals −a [35]. Figure 1 illustrates the flowchart of our proposed method.

### 2.3. Statistical Analysis

The demographic and clinical measures between MDD and HC individuals were evaluated using one-way analysis of variance (ANOVA) for continuous variables and chi-square tests for categorical variables.

As mentioned before, the majority of findings in the literature regarding MDD patients focus on theta and alpha frequency bands. Additionally, our hypothesis aims to evaluate both electrode and source levels. Therefore, we initially calculated PSD and periodic spectra for theta and alpha frequencies at the electrode level across all electrodes in five conventional brain areas, including frontal (Fpz, Fp1, Fp2, AF3, AF4, Fz, F1, F2, F3, F4, F5, F6, F7, F8, FCz, FC1, FC2, FC3, FC4, FC5, FC6, FT7, FT8), central (Cz, C1, C2, C3, C4, C5, C6, CPz, CP1, CP2, CP3, CP4, CP5, CP6), temporal (T7, T8, TP7, TP8), parietal (Pz, P1, P2, P3, P4, P5, P6, P7, P8, POz, PO3, PO4, PO5, PO6, PO7, PO8), and occipital (Oz, O1, O2) regions. The values for each brain region were then computed by averaging the electrode-level results within the respective region.

To analyze EEG data from various brain regions in the source-level, we utilized exact Low-Resolution Electric Tomography (eLORETA) to estimate the cortical source activity underlying scalp-recorded EEG signals [47]. In addition to eLORETA, several methods have been proposed for estimating cortical source activity from EEG data, including LORETA, standardized LORETA (sLORETA), minimum norm estimation (MNE), and beamforming. eLORETA offers significant advancements over earlier tomographic techniques like LORETA and sLORETA. In the presence of structural noise, this method has no spatial bias in source localization and allows for accurate localization of deep cortical sources [47,48]. Moreover, eLORETA is better at controlling false positives [49] and has a zero peak localization error [50] compared to other methods, including MNE and beamforming. eLORETA tackles the challenge of the EEG inverse problem by providing linear estimates of neural current density across cortical voxels within a defined head volume conductor model. This technique calculates solutions for each frequency band of the EEG, using the data collected from scalp electrodes [47]. The result is an estimate of neural current density at specific current dipoles, distributed across 6239 voxels (with a 5 mm resolution) in the cortical source space, focusing on the cortical gray matter. eLORETA specifically estimates local neural currents along three axes (‘z’, ‘x’, and ‘y’) within each voxel. This approach allows for estimating source activities from EEG rhythms in specific Regions of Interest (ROIs) [51]. The selection of these ROIs focuses on five main brain regions. The model used is based on a realistic brain shape from the Montreal Neurological Institute (MNI152 template), a commonly adopted standard in neuroimaging research [51].

Subsequently, we generated aperiodic spectra across the aforementioned five brain regions for both electrode and source levels in HC and MDD participants. A Wilcoxon rank-sum test was used to evaluate the differences in the extracted features, including periodic and aperiodic components, as well as PSD, between healthy and MDD individuals because the data did not follow a normal distribution. The significance level was α = 0.05 for all tests. Then, post hoc analyses with Bonferroni correction were applied to control for the effect of 50 comparisons ([2 conditions (PSD, periodic) × 2 spaces (electrode, source) × 5 brain regions × 2 frequencies (theta, alpha)] + [1 condition (aperiodic) × 2 spaces (electrode, source) × 5 brain regions]). In addition, we calculated Hedge’s g effect size (ES) for each comparison to better evaluate our findings.

We evaluated the statistical results of each periodic, aperiodic, and PSD feature, selecting those with the highest ESs and the corresponding lowest *p*-values. These selected features were then used in logistic regression to classify HC and MDD individuals. Additionally, we combined these features to determine the most effective set for diagnostic purposes. The features were combined in the following ways:

1. Combination by frequency band: we paired features from the same frequency band to form the feature sets.
2. Combination by analysis type: we paired features from the same analysis type to form the feature sets.
3. Dual-component combination: we combined features from different components of our analysis (periodic + aperiodic) to form the feature sets.

We then employed the Leave-One-Subject-Out (LOSO) approach to assess the performance of the models. In this approach, the data from one subject are reserved as the test set while the model is trained on the remaining subjects. This process is repeated for each subject in the same manner. The overall cross-validation performance is evaluated using the Area Under the Curve (AUC). The pointwise confidence intervals are obtained through bootstrapping with random sub-sampling (n = 1000). To identify the best set of features, we evaluated each combination based on AUC. The combination with the highest performance across this metric was selected as the best set for diagnostic purposes.

Finally, we conducted Spearman’s correlations, a non-parametric test, between the selected features with the highest ESs for periodic, aperiodic, and PSD components, as well as the BDI and its subscales, namely anhedonia and melancholia. It is worth noting that a higher BDI score indicates higher severity in depression symptoms. The anhedonia subscale assesses symptoms related to the loss of interest or pleasure in activities, diminished ability to feel pleasure, and reduced motivation [52]. The melancholia subscale in the BDI typically assesses these specific symptoms related to melancholic depression, characterized by severe symptoms such as pervasive sadness, anhedonia, psychomotor disturbances [52].

## 3. Results

All the analyses, including statistical tests, machine learning procedures, and correlation analyses, were performed using MATLAB 2019a software (MathWorks, Inc., Natick, MA, USA) on a Windows PC with a 2.50 GHz Intel^®^ Core™ i5-10300H processor and 16 GB of RAM.

### 3.1. EEG Power Comparisons in Different Brain Regions

**Figure 2:**
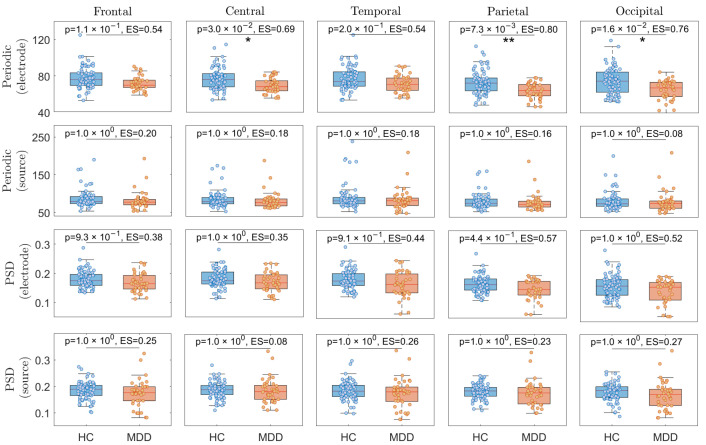
The theta periodic and PSD analyses for HC vs. MDD individuals within each brain region separately. * and ** represent Bonferroni corrected *p*-values of less than 0.05 and 0.01, respectively. ES stands for effect size.

Figure 2 displays the theta periodic and PSD analyses comparing HC and individuals with MDD across different brain regions. Specifically, the first and second rows present results for the theta periodic components, with the first row focusing on electrode-level analysis and the second row on source-level analysis. Additionally, the third and fourth rows of Figure 2 compare theta power based on PSD, where the third row corresponds to electrode-level analysis and the fourth row to source-level analysis. As can be observed in Figure 2, the theta periodic component in the electrode-level condition shows significant differences between the two groups across all brain regions, with the highest Hedge’s g ES in the parietal lobe (*p* = 7.3 $\times \;10^{-3}$, Hedge’s g ES = 0.80) Additionally, the second highest ES is for the theta periodic component in the electrode-based occipital brain regions (*p* = 1.6 $\times \;10^{-2}$, Hedge’s g ES = 0.76). There are no significant differences in the PSDs of the five brain lobes for both source- and electrode-level measurements.

**Figure 3:**
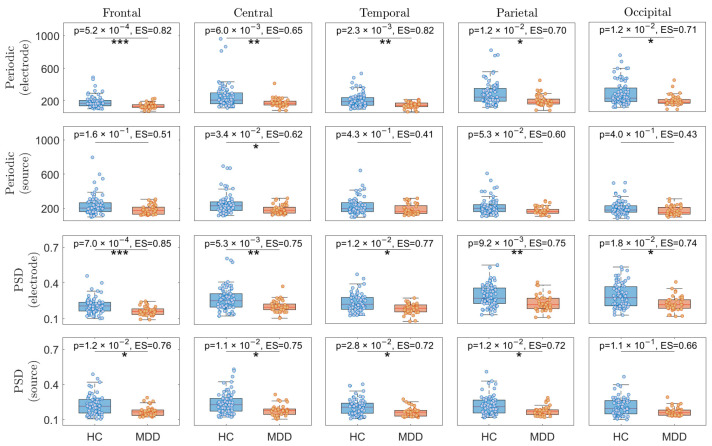
The alpha periodic and PSD analyses for HC vs. MDD individuals within each brain region separately. *, **, and *** represent Bonferroni corrected *p*-values of less than 0.05, 0.01, and 0.001, respectively. ES stands for effect size.

The alpha periodic and PSD analyses for the HC and individuals with MDD for each brain region are illustrated in Figure 3. It is worth noting that the first and second rows show the results for the alpha periodic component, with the first row related to electrode-level analysis and the second row related to source-level analysis. Similarly, the third and fourth rows of Figure 3 compare the results for alpha PSD. The third row pertains to electrode-level analysis, while the fourth one refers to source-level analysis. The Bonferroni correction was applied to adjust all the *p*-values. Significant differences were observed in all the brain regions between HC and MDD participants. The frontal lobe showed the highest Hedge’s g ES for both alpha periodic and PSD in electrode-level analysis (Alpha periodic: *p* = 5.2 $\times \;10^{-4}$, Hedge’s g ES = 0.82; Alpha PSD: *p* = 7 $\times \;10^{-4}$, Hedge’s g ES = 0.85).

**Figure 4:**
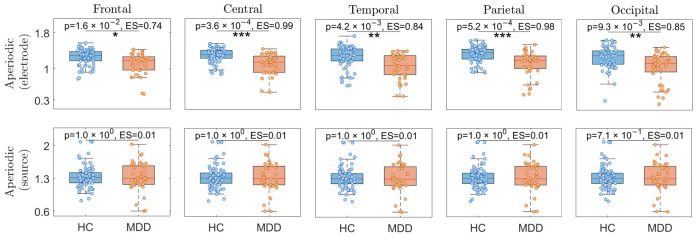
The aperiodic analyses for HC vs. MDD individuals within each brain region separately. *, **, and *** represent Bonferroni corrected *p*-values of less than 0.05, 0.01, and 0.001, respectively. ES stands for effect size.

The comparison for the aperiodic components between two groups for both electrode- and source-level conditions across all five brain regions is illustrated in Figure 4. The first row is related to electrode-level analysis, and the second row is related to source-level analysis. As observed from this figure, all five brain regions show significant differences between the two groups in aperiodic electrode-level conditions, whereas there are no significant differences between the two groups in aperiodic source-level conditions. Additionally, the two highest Hedge’s g ES values were observed in the central and parietal lobes. (Central aperiodic: *p* = 3.6 $\times \;10^{-4}$, Hedge’s g ES = 0.99; parietal aperiodic: *p* = 5.2 $\times \;10^{-4}$, Hedge’s g ES = 0.98). Given the closely similar ES values observed in both regions for aperiodic components, the parietal region was selected for further analysis due to its greater relevance in studies of depression.

### 3.2. Classification

**Figure 5:**
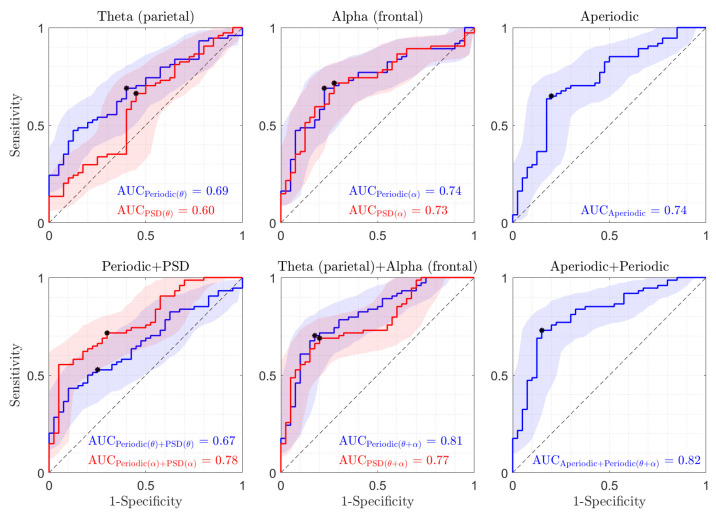
Results of logistic regression-based classifications using parietal theta and frontal alpha for both the periodic component and PSD, as well as parietal aperiodic separately and in combination. The results of the best classifier are indicated with a star.

Based on the findings from the previous section, our focus was on evaluating parietal theta and frontal alpha using both periodic components and PSD under electrode-level conditions. To achieve this, we employed a logistic regression model to classify groups into HC versus MDD individuals using the specified features both individually and in combination. Additionally, separate logistic regression models were used to classify the two groups using the parietal aperiodic component, as well as a combination of both parietal aperiodic and periodic components (including parietal theta and frontal alpha), all under electrode-level conditions. Figure 5 presents the results of logistic regression-based classifications. The upper left panel of Figure 5 shows the classification results comparing the frontal alpha periodic component with PSD. In contrast, the upper middle panel compares the parietal theta periodic component and PSD. Additionally, the upper right panel illustrates the classification results for the aperiodic component in the parietal lobes. The lower left panel of Figure 5 displays the classification results for the combination of PSD and periodic components in both theta and alpha frequency ranges. The lower middle panel illustrates the combination of parietal theta and frontal alpha in both PSD and periodic components. Finally, the lower right panel combines periodic parietal theta, frontal alpha, and parietal aperiodic components.

### 3.3. Correlation Analysis

**Figure 6:**
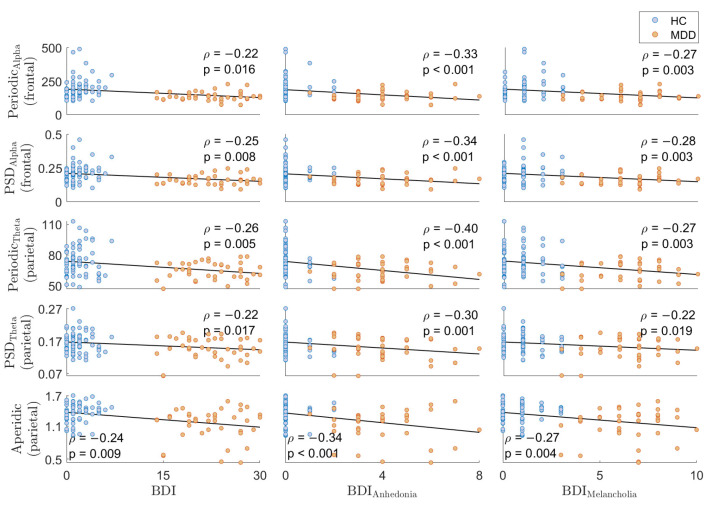
Correlation of frontal alpha periodic (**first row**), frontal alpha PSD (**second row**),parietal theta periodic (**third row**), parietal theta PSD (**fourth row**), and parietal aperiodic component (**fifth row**) with the BDI and its subscales. It is worth noting that the BDI subscales are anhedonia and melancholia. ρ indicates Spearman’s rho.

Given that the most significant differences are found in frontal alpha and parietal theta power in both the periodic component and PSD, as well as in the aperiodic component in the parietal lobe (all in electrode-level conditions), we assessed the association between presence and severity of depression symptoms using BDI and its subscales and these EEG metrics. Figure 6 illustrates the correlation of alpha periodic (first row) and alpha PSD (second row) in the frontal lobe with the BDI and its subscales, including anhedonia and melancholia. Furthermore, Figure 6 shows the correlation of theta periodic (third row), theta PSD (fourth row), and the aperiodic component (fifth row) in the parietal lobe with the BDI and its subscales. From the correlation analysis results, it is evident that all the mentioned metrics correlate negatively with the BDI scores and their subscales. The best correlation results are between periodic parietal theta and the anhedonia BDI subscale (ρ = −0.40, *p* = 1.01 $\times \;10^{-5}$).

## 4. Discussion

The current study is the first to investigate the periodic and aperiodic components of EEG in comparison with conventional PSD under both electrode- and source-level conditions in different brain regions, aiming to differentiate between HC and MDD individuals. Initially, both groups were compared across all five brain regions. Then, the most significant results were used for further analysis, including classification and correlation analysis. It should be noted that, based on the main findings in MDD patients [4,5,23,24,25,26,27,28,29,30,31], our focus is on the theta and alpha frequency bands.

When examining the theta frequency band, the comparisons of EEG power across five different brain regions indicate that the highest ES is observed in the parietal lobe under electrode-level conditions. Recently, Mazza et al. demonstrated that alterations in depression involve both aperiodic broadband and periodic theta components by utilizing simulated resting-state activity [53]. Despite inconsistencies in the abnormality of theta brain waves in MDD patients compared to HC, our study aligns with some research that found a decrease in theta activity [54]. This decrease may indicate a connection between theta activity and cerebral metabolism in the anterior cingulate cortex (ACC), along with a functional disconnection in the frontocingulate pathways, which alters emotional processing capacity [55,56].

On the other hand, when analyzing the alpha frequency band across two groups and in the five main brain regions, the highest ES is found in the frontal lobe under electrode-level conditions. In this scenario, the results from the periodic component and PSD align closely, with PSD demonstrating slightly better outcomes. These findings are consistent with some EEG [25,31] and magnetoencephalography (MEG) [57] studies that found that individuals diagnosed with MDD exhibited a decrease in alpha activity compared to the HC group. They replicated the finding that alpha activity in the frontal and subcortical regions of MDD patients may indicate cognitive impairments related to emotion regulation, psychomotor retardation, and the processes related to motivation and reward [58,59]. It is possible that EEG alpha power reflects changes in excitatory and inhibitory pathways in depression through the generalized effects of brain-derived neurotrophic factor (BDNF) on cortical excitability processes [25,60]. On the other hand, reduced alpha activity can be associated with increased brain activation or attentional demands, suggesting that individuals with depression might experience a more disrupted or engaged brain state compared to HC. This alternation has also been observed in other types of depression, such as bipolar disorders [61,62]. It is noteworthy that the similarity in findings between MDD and bipolar depressive disorders may be due to the overlap of symptoms [63]. Additionally, a patient initially diagnosed with MDD might later have a manic or hypomanic episode, leading to a re-diagnosis of bipolar disorder, which includes both depressive and manic/hypomanic episodes [63].

Evidently, the most significant results are in the frontal and parietal brain regions, which have been widely reported in previous studies related to depressive disorders. The parietal lobe, with its involvement in processing sensory information, spatial awareness, decision making, and speech comprehension, contributes to the perception and integration of external stimuli [64], which can be altered in MDD. Additionally, the frontal lobe, crucial for executing functions such as planning, decision making, and working memory, is integral to managing complex cognitive tasks [65]. The frontal lobe, particularly the prefrontal cortex, is also vital for emotional regulation, social behavior, impulse control, and personality expression [65]. Dysfunctions in these areas can lead to the impaired cognitive and emotional processing observed in MDD.

It should be stressed that the validation of source-level analysis remains challenging in EEG studies. Achieving a high level of accuracy in EEG requires many electrodes with precise placements, consideration of head shapes, and detailed brain-forward models, which also include precise estimates of tissue conductivities [40]. Therefore, based on the above limitations of source-level analysis and for the reproducibility of some approaches, like PSD, electrode-level analysis yields more reproducible and reliable results [66].

Our machine learning results reveal that the periodic components perform slightly better than PSD in distinguishing between MDD and HC when using alpha and theta features individually. Notably, when using just one feature, both the alpha periodic and aperiodic components exhibit significantly superior performance compared to others, each achieving an AUC of 0.74. It is noteworthy that our results suggest that the alpha periodic component may provide a more discriminative feature compared to the theta periodic component. Furthermore, combining the periodic components of both theta and alpha leads to significantly better classification results compared to using PSD features alone. The best classification results are achieved using periodic and aperiodic features (only three features), resulting in an AUC of 0.82 for distinguishing between the two groups.

Finally, the correlation analysis shows negative correlations between decreased both periodic parietal theta and frontal alpha activities and elevated scores on the BDI and its subscales. While the correlations between features and BDI and melancholia subscale scores are generally low, the relatively higher correlations observed with the anhedonia subscales suggest that parietal theta may be a more relevant neural marker for anhedonia. By correlating parietal theta activity (particularly within periodic components) with the anhedonia subscales, we provide further evidence supporting the utility of these features in understanding the neural underpinnings of depression [67]. These results contribute to the growing evidence that the periodic theta and alpha in addition to aperioidc components of EEG are important in understanding brain activity related to depressive symptoms, especially anhedonia.

However, there are some limitations to this study that should be addressed in future research. Future studies should include a larger number of participants in both groups and consider combining multiple datasets and apply a single machine learning model to improve generalizability. Furthermore, to reduce gender bias and improve the generalizability of results, future studies should aim to include approximately equal numbers of participants from each gender. Additionally, given the paradoxical findings in neuroimaging literature related to MDD patients, future research should apply stricter criteria to differentiate groups based on factors such as the duration of untreated illness (DUI), genetic risks, and medication history to validate results more effectively. Therefore, further investigations are required to better understand the underlying pathophysiological mechanisms of this disorder and to identify the most effective medications, such as Selective Serotonin Reuptake Inhibitors (SSRIs) or mood stabilizers, for treating mood disorders [68].

## 5. Conclusions

The findings of this study provide novel insights into the neural correlates of MDD by examining both the periodic and aperiodic components of resting-state EEG across different brain regions. By distinguishing between these components, we addressed a significant gap in the literature, where previous research often failed to separate the aperiodic component from the EEG spectrum in both the electrode- and source-level conditions, potentially masking critical differences in the brain activity associated with MDD. Our results demonstrate that separating periodic from aperiodic components can enhance the accuracy of classifying MDD and HC individuals, highlighting the importance of focusing on specific EEG features. Our results also showed that electrode-level analysis yields higher AUC values compared to source-level analysis, indicating that surface-level EEG signals provide more discriminative information. This study also underscores the relevance of frontal alpha and parietal theta activity, particularly in their association with depressive symptoms, including anhedonia. By correlating these EEG metrics with the BDI scores and subscales, we provide further evidence supporting the utility of these features in understanding the neural underpinnings of depression. Overall, our research contributes to a more nuanced understanding of EEG abnormalities in MDD and offers a potential pathway for improved diagnostic and therapeutic approaches by emphasizing the distinct roles of electrode-level periodic and aperiodic EEG components. 

## Figures and Tables

**Table 1:** Demographic characteristics and scores from BDI subscales (anhedonia: BDI_Anh, melancholia: BDI_Mel) and Trait Anxiety Inventory (TAI).

	HC (n = 74)	MDD (n = 40)	*F*/$\chi^{2}$*(df)*	*p*-Value
**Age in years (SD)**	18.98 (1.21) *	18.70 (1.15)	1.47 (1, 111)	0.22
**Gender (male/female)**	35/39	10/30	5.40 (1)	0.02
**BDI**	1.71 (1.65)	22.27 (4.69)	1.15e3 (1, 112)	<0.001
**BDI_Anh**	0.16 (0.46)	4 (1.58)	3.75e2 (1, 112)	<0.001
**BDI_Mel**	0.83 (0.90)	6.42 (1.72)	5.16e2 (1, 112)	<0.001
**TAI**	31.12 (5.49)	56.15 (6.81)	4.53e2 (1, 112)	<0.001

* Mean (SD) based on 73 participants. Age was missing for one participant. SD refers to standard deviation.

## Data Availability

The dataset used in this study is publicly available at http://predict.cs.unm.edu/downloads.php (accessed on 31 August 2023). Furthermore, the data were pre-processed using the EEGLAB toolbox (https://sccn.ucsd.edu/eeglab/index.php (accessed on 31 August 2023).) in MATLAB 2019a (MathWorks, Inc., Natick, MA, USA). The processing code can be obtained from the corresponding author upon reasonable request.

## Abbreviations

The following abbreviations are used in this manuscript:

ACC	Anterior cingulate cortex
ANOVA	One-way analysis of variance
AUC	Area Under the Curve
BDI	Beck Depression Inventory
BDNF	Brain-derived neurotrophic factor
DUI	Duration of untreated illness
EEG	Electroencephalography
eLORETA	exact Low-Resolution Electric Tomography
ES	Effect size
GABA	Gamma-aminobutyric acid
HAPPE	Harvard Automated Preprocessing Pipeline
HC	Healthy control
LOSO	Leave-One-Subject-Out
MARA	Multiple Artifact Rejection Algorithm
MEG	Magnetoencephalography
MDD	Major depressive disorder
MNE	Minimum norm estimation
PSD	Power spectral density
ROIs	Regions of Interest
sLORETA	standardized Low-Resolution Electric Tomography
SSRIs	Selective Serotonin Reuptake Inhibitors
TAI	Trait Anxiety Inventory
W-ICA	Wavelet-enhanced independent component analysis
